# Psychometric assessment of Persian translation of Yale Food Addiction Scale Version 2.0 (YFAS 2.0) in Iranian college students

**DOI:** 10.1186/s40337-022-00689-5

**Published:** 2022-11-10

**Authors:** Nikzad Ghanbari, Roghieh Nooripour, Abbas Firoozabadi, Tabassom Saeid Par Var, Pamela Wisniewski, Seyed Ruhollah Hosseini

**Affiliations:** 1 Department of Clinical Psychology, Faculty of Psychology and Educational Sciences, Shahid Beheshti University, Tehran, Iran; 2 Department of Counseling, Faculty of Education and Psychology, Alzahra University, Tehran, Iran; 3 Department of Psychology, Faculty of Education Sciences and Psychology, Ferdowsi University of Mashhad, Mashhad, Iran; 4 Addiction Department, School of Behavioral Sciences and Mental Health, Iran University of Medical Sciences, Tehran, Iran; 5 Department of Computer Science Faculty Fellow, Flowers Family in Engineering, Vanderbilt University, Nashville, TN USA

**Keywords:** Food addiction, Psychometric properties, Students, Validation, YFAS 2.0

## Abstract

**Background:**

Food addiction at the individual level causes physical and mental health problems, impairs individuals' social functioning, and causes dysfunction in the family system. Therefore, a tool to identify this behavioral disorder is one of the health requirements of communities. This research aimed to investigate the psychometric assessment of the Persian translation of Yale Food Addiction Scale Version 2.0 (YFAS 2.0) in Iranian college students.

**Method:**

This research was cross-sectional descriptive, and 451 students were selected by convenience sampling method. Yale Food Addiction Scale Version 2.0 (YFAS 2.0), Depression, Anxiety, and Stress Scale-21 (DASS-21), and Food Craving Questionnaire-Trait, reduced (FCQ-T-r) were used to collect data.

**Results:**

The confirmatory factor analysis indicated that single-factor model provides a good fit to data (SRMR = 0.078; CFI = 0.94; NFI = 0.92; IFI = 0.94; RFI = 0.91; GFI = 0.90; RMSEA = 0.078). The YFAS's 2.0 positive correlations with three DASS-21 subscales ranged from 0.30 to 0.39, and Food Craving Questionnaire-Trait, reduced (FCQ-T-r) ranged from 0.58 to 0.72. All correlations were statistically significant, indicating acceptable convergent validity (*P* < 0.01).

**Conclusion:**

The validity of the Persian questionnaire translation has been confirmed. Researchers and specialists can use this scale to diagnose food addiction for research or diagnostic purposes in Iranian society.

## Plain English summary

The term "food addiction" refers to the behavior of consuming highly palatable foods (such as salty, fatty, and sweet foods) in quantities beyond what is necessary to maintain a healthy diet. Some individuals may become addicted to food by consuming palatable and highly processed foods (e.g., fast food). There is growing scientific interest in food addiction. This study aims to examine the psychometric assessment of Persian translation of Yale Food Addiction Scale Version 2.0 (YFAS 2.0) In Iranian college students. Participants in the study included 451 Iranian college students who completed both questionnaires online. The results showed that the YFAS's 2.0, DASS-21, and FCQ-T-r had positive correlations indicating acceptable convergent validity. So the Persian translation YFAS 2.0 is suitable for measuring food addiction among Iranian college students, and it could be used in clinical and research settings.

## Introduction

Obesity and overweight are among many countries' leading health problems and primary metabolism diseases. The emergence and development are influenced by various social, behavioral, cultural, physiological, genetic, and metabolic [1]. Obesity is one of the most preventable causes of death worldwide, with rising rates among adolescents and children [2]. Overeating is the leading cause of obesity or overweight, and its prevalence has increased dramatically worldwide. Research has shown that addictive eating behaviors can be a factor that can lead to overeating and obesity [3]. Subgroups of overweight and obese people describe themselves as "addicted" to food, characterized by emotional eating, impaired control over their eating habits, and food cravings.

The neurological foundations of food addiction have several similarities with substance use and drug abuse disorders. However, recognizing “food addiction as a clinical disorder” is still controversial [4]. Evidence has been found in several similarities between food addiction and drug addiction, strongly supporting the concept of food addiction [5]. Food addiction includes cravings, drinking, and overeating [6]. Also, behavioral symptoms associated with food dependence appear in patients with abnormal anorexia nervosa, eating disorders, and obesity, even in the non-clinical population [7]. Numerous diagnostic criteria and methods have been proposed to assess food addiction [8]. Interviews/questionnaires may yield a food addiction diagnosis if three of the following seven symptoms are present during the past year. These seven signs are: (1) despite feeling full, the individual often craves certain foods. (2) When a person starts eating, craving, and often feels consumed much more than intended. (3) Individual eats with cravings and sometimes feel overeating. (4) The person often feels guilty after eating certain foods and will start eating again soon after eating. (5) Although individuals know that certain foods cause physical harm, including weight gain, they feel unable to control unhealthy food consumption. (6) A person has repeatedly tried to crack or regulate certain foods but has failed. (7) The individual often hides unhealthy foods from others [9, 10].

Food addiction in people could be diagnosed based on a person’s behavioral symptoms and eating habits. So, the existence of a tool to identify this behavioral disorder is one of the health requirements of communities. If it is determined that the cause of some problems and diseases is food addiction, it can be used on a large scale to make changes and reforms in the food industry and eliminate some foods [11].

Research has led to the development of a tool that can identify individuals who show symptoms of "dependence" on certain foods. Gearhardt and colleagues [12] developed the Yale Food Addiction Scale (YFAS) in 2009, which measures addictive-like eating behaviors based on diagnostic criteria for substance dependence. Substance-related and addictive disorders (SRAD) significantly changed the Diagnostic and Statistical Manual of Mental Disorders (DSM-5). This revised version of the YFAS(YFAS 2.0) maintains consistency with current diagnostic understandings of food addiction while improving its psychometric properties [13]. Yale Food Addiction Scale Version 2.0 (YFAS 2.0) is one widely used scale developed to identify food addiction characteristics and has internationally been validated by several studies [14, 15]. Also, some changes have already taken place in YFAS 2.0, including a modified version of YFAS 2.0 and a version for assessing children. Pepino, Stein, Eagon, and Klein [16] used YFAS 2.0 in a clinical sample of bypass surgery before and after surgery (n = 44) and reported the prevalence of food addiction at nine months, starting at 32% and 2%.

One of the main problems in measuring food addiction has been the lack of questionnaires or standard and usable self-report tools in the Persian language. Due to societies’ cultural-social and economic differences, it is necessary to evaluate its validity and reliability before using it. These findings indicate that this scale is appropriate for assessing food addiction. Given the rapid growth of food addiction studies, it seems necessary to identify proper tools to evaluate the number of addictive foods. The validity of YFAS 2.0 has not been evaluated among students in Iran yet. Therefore, this study evaluates the psychometric assessment of the Persian translation of Yale Food Addiction Scale Version 2.0 (YFAS 2.0) among Iranian college students.

## Methods

This research is cross-sectional descriptive, and its statistical population was all Ferdowsi University of Mashhad students. The data collection period began in June 2018 and January 2019.

The inclusion criteria included lack of metabolic diseases (such as diabetes) and lack of dietary supplements in this study. Exclusion criteria were using psychotropic medication and failure to complete the questionnaire. Alongside the translated YFAS 2.0, the other scales used in this study were translated into Persian.

## Participants

Before the survey, informed consent was obtained from participants. This study strictly protected participants' confidentiality. The data collection method included collecting the answers to the research questionnaires via the internet in Iran. Consenting participants and questionnaires were then entered into *Google Forms,* and the link was sent to social networks to be completed online by the respondents. The survey also included an information sheet reminding participants of their voluntary and anonymous participation, and it is coded to do the test–retest reliability step. The respondents had the right to choose whether to join the study and provide information or withdraw—inter-rater agreement measured by Cohen’s kappa coefficient (K = 0.76) indicates acceptable agreement.

451 Iranian students participated in this study and were asked to complete questionnaires. The participants’ mean age was 25.8 years (SD = 9.3), and the age range was 17–60.

## Measures

### Yale Food Addiction Scale Version 2.0 (YFAS 2.0)

We used a 35-item Yale Food Addiction scale 2.0 [13], a well-known food dependency assessment, to determine food addiction symptoms over 12 months ago. These symptoms include tolerance, reversal, overdose, persistent tendency or an unsuccessful attempt to reduce it, spending too much time or recycling, and continued use despite being aware of the consequences and activities due to abandoned materials. The scale also assesses clinical disorders or discomfort resulting from addictive eating habits. The YFAS 2.0 are an eight-point Likert scale that varies from “never” (= 0) to “every day.” (= 7). A question is considered positive if its score is equal to or higher than the threshold, depending on whether the point is higher or lower than that threshold. Cronbach’s alpha coefficient of 0.87 indicates an adequate internal coefficient [17].

The Depression, Anxiety, and Stress Scale-21 (DASS-21) was applied to assess the anxiety and stress scale. Lovibond and Lovibond developed the 21-item scale in 1995 to measure stress, anxiety, and depression. Each item’s scoring method rose from zero (it does not apply to me or never) to 3 (often applied to me). The sub-scales and related items were as follows: anxiety (items 2, 4, 7, 9, 15, 19, 20), stress (items 1, 6, 8, 11, 12, 14, 18), and depression (items 3, 5, 10, 13, 16, 17, 21). Brown, Chorpita, Korotitsch, and Barlow [18] also reported validity of 0.77 for the scale. Each component contained seven items, whose final score could be obtained by summing the items’ scores. Thus, the score could range between 0 and 21 per subscale. In Iran, the reliability of the DASS-21 has been reported at 0.82 via Cronbach’s alpha method [19].

### Food Craving Questionnaire-Trait, Reduced (FCQ-T-r)

It was designed and developed by Cepeda-Benito et al. [20] to measure adult food-trait cravings. This questionnaire contains 15 items. Answers were recorded on a Likert scale from one (never not applicable or not applicable) to six (always).

### Procedure

An ethical committee approved the study procedure of Ferdowsi University of Mashhad (IR/09/11/1398), and informed consent was obtained from the participants. All methods have been carried out under relevant guidelines and regulations.

This research was divided into two phases: the instrument's translation, its psychometric properties analysis, and its validity verification. The YFAS 2.0 was translated into Farsi (Persian) using the back-translation technique in the first phase. In this technique, one translation team translates the scale into Persian, and then the second team translates it back into the original language. The translation accuracy was judged by closely matching the second team’s original version. However, as Hambleton et al. [21] pointed out, this commonly used technique has shortcomings. They suggested that translators be proficient in both languages and familiar with both cultures. The quality of the translation has been assessed as to how it fits the initial text. Accordingly, two translators were contacted to help with the study. The translators worked independently, and no significant differences were found in the translation and expression of the items. The authors subsequently reached a consensus with the translators on both versions. Finally, some items were revised by a professor of English and other psychologists to make them more understandable and comprehensible to the target audience. Care was taken to ensure that the length of the items corresponded to the original scale. The authors then achieved agreements with the translators for the final version.

### Statistical analysis

In the first stage, the outliers were checked by z—scores and box plots (values + 3 standard deviations from the mean indicate univariate outliers). A visual check, skewness, and Kurtosis values demonstrated normally distributed data (see Table 1). In the second place, descriptive statistics, including mean, Standard Deviation (SD), and range, were calculated for Yale Food Addiction Scale Version 2.0 (YFAS 2.0). Third, Confirmatory factorial analysis (CFA) for ordinal data was used to investigate the internal structure of the YFAS 2.0. Maximum Likelihood was used for the estimation method, and Fitness indexes were evaluated with a 90 percent confidence interval for Model fitness, including Root Mean Square Error of Approximation (RMSEA), Parsimony Normed Fit Index (PNFI), Comparative Fit Index (CFI), Incremental Fit Index (IFI), Relative Fit Index (RFI), Standardized Root Mean Square Residual (SRMR) and Normed Fit Index (NFI). For a good fit model, CFI, IFI, RFI, NFI should be greater than 0.90, AGFI greater than 0.80, PNFI greater than 0.50, RMSEA less than 0.08, and SRMR less than 0.09. Fourth, Cronbach’s alpha coefficient of the YFAS 2.0 was estimated. Furthermore, in fifth place, Pearson’s correlation between the YFAS 2.0 with FCQ-T-r’s subscales and DASS-21 was investigated to obtain evidence of validity with other variables. For the data analysis, the SPSS-26 and LISREL 8.8 statistical programs were used.

**Table 1:** Descriptive statistics of the Yale Food Addiction Scale Version 2.0 (YFAS 2.0) in the sample of male and female university students

	Mean	SD	Kurtosis	Skewness	Range	Alpha
Male	68.15	32.03	.688	1.14	155	.935
Female	62.08	29.23	.928	2.68	159	.939

### Preliminary analysis

Preliminary tests, such as data loss analysis, discarded data, and normality of the data were performed before the CFA. Discarded data were assessed by the Mahalanobis distance square with a significance level of 0.001 in AMOS software, and no discarded pieces of data were identified. Skewness (0.154–0.181) and Kurtosis (0.35–2.15) were used to test the normality assumption of the items. The normality of the data is met when the values range between ± 2 for skewness and ± 3 for Kurtosis [22]; the results showed the normal distribution of the data.

## Results

### Descriptive statistic

The participants’ ages ranged from 17 to 60 (M = 25.8 years, SD = 9.29). Regarding gender status, 293 (65.0%) were female, and 158 (35.0%) were male. 352 (78.0%) were single, and 99 (22.0%) were married. 422 (93.6%) of the sample had no psychiatric history, 26 (5.8%) had a psychiatric history, and 3 (0.7%) did not report this part. Regarding medical history status, 396 (87.8%) of the sample had no medical history, 50 (11.1%) had a medical history, and 5 (1.1%) did not report their medical status. Regarding educational status; 188 (41.7%) high school diploma, 210 (46.6%) bachelor, 40 (8.9%) master and 13(2.9%) PhD. The mean and standard deviation (SD) for Yale Food Addiction Scale Version 2.0 (YFAS 2.0) were 64.63 and 30.65, respectively (see more in Table 1).

#### Confirmatory factor analysis (CFA)

The CFA findings for a single factor structure are illustrated in Table 2. These results are acceptable, given that the factor loadings for all items are significant.

**Table 2:** Standardized factor loadings for one-factor solution of the Yale Food Addiction Scale Version 2.0 (YFAS 2.0) identified using confirmatory factor analysis and Descriptive statistics for all YFAS 2.0 items

In the past 12 months…	Items statistics			Item-total statistics		
	M	SD	F.L	V	I.T	C.D
When I started to eat certain foods, I ate much more than planned	3.527	2.074	0.36	885.78	.400	.933
I continued to eat certain foods even though I was no longer hungry	2.851	2.075	0.37	884.12	.413	.933
I ate to the point I felt physically ill	1.434	1.140	0.51	904.36	.492	.932
I worried a lot about cutting down on certain types of food, but I ate them anyways	2.516	1.942	0.54	871.39	.560	.931
I spent a lot of time feeling sluggish or tired from overeating	1.913	1.627	0.49	884.91	.536	.931
I spent a lot of time eating certain foods throughout the day	2.084	1.684	0.47	887.88	.485	.932
When certain foods were unavailable, I went out of my way to get them. For example, I went to the store to get certain foods even though I had other things to eat at home	2.385	1.825	0.59	885.26	.468	.932
I ate certain foods so often in such large amounts that I stopped doing other important things. These things may have been working or spending time with family or friends	1.443	1.134	0.46	900.40	.554	.931
I had problems with my family or friends because I was overrated	1.884	2.589	0.67	866.19	.436	.934
I avoided work, school, or social activities because I was afraid I would overeat there	1.374	1.156	0.64	896.51	.600	.931
I felt irritable, nervous, or sad when I cut down on or stopped eating certain foods	1.874	1.557	0.54	879.50	.623	.930
If I had physical symptoms because I hadn’t eaten certain foods, I would eat those foods to feel better	1.957	1.514	0.61	887.99	.544	.931
If I had emotional problems because I hadn’t eaten certain foods, I would eat those foods to feel better	2.160	1.684	0.61	876.77	.600	.931
I had physical symptoms when I cut down on or stopped eating certain foods. For example, I had headaches or fatigue	1.697	1.439	0.29	887.71	.579	.931
In the last 12 months, when I cut down or stopped eating certain foods, I had intense cravings for them	2.131	1.678	0.31	903.25	.331	.933
My eating behavior caused me a lot of distress	1.763	1.504	0.31	904.80	.357	.933
I had significant problems in my life because of food and eating. These may have been problems with my daily routine, work, school, friends, family, or health	1.494	1.221	0.29	911.33	.361	.933
I felt so bad about overeating that I didn’t do other essential things. These things may ha**ve** been working or spending time with family or friends	1.437	1.147	0.31	914.44	.341	.933
My overeating got in the way of me taking care of my family or doing household chores	1.347	1.110	0.26	915.56	.337	.933
I avoided social situations because people would **dis**approve of how much I ate	1.366	1.087	0.71	918.27	.303	.933
I avoided social situations because people would **dis**approve of how much I ate	1.419	1.256	0.65	889.56	.644	.931
I kept eating the same way even though my eating caused emotional problems	1.734	1.660	0.57	876.60	.611	.930
I kept eating the same way even though my eating caused physical problems	1.798	1.839	0.62	877.62	.536	.931
Eating the same amount of food did not give me as much enjoyment as it used to	1.768	1.527	0.64	883.68	.588	.931
I wanted to cut down on or stop eating certain foods, but I couldn’t	2.179	1.890	0.64	865.79	.630	.930
I needed to eat more and more to get the feeling I wanted from eating. This included reducing negative emotions like sadness or increasing pleasure	1.815	1.616	0.71	869.17	.710	.929
I didn’t do well at work or school because I **overate**	1.474	1.267	0.56	890.00	.632	.931
I kept eating certain foods even though I knew it was physically dangerous. For example, I kept eating sweets even though I had diabetes. Or I kept eating fatty foods despite having heart disease	1.791	1.532	0.70	887.87	.539	.931
I had such strong urges to eat certain foods that I couldn’t think of anything else	1.596	1.382	0.71	883.94	.651	.930
I had such intense cravings for certain foods that I felt **I had to eat them immediatel**y	1.816	1.483	0.49	884.97	.592	.931
I tried to cut down on or not eat certain kinds of food, but I wasn’t successful	2.071	1.789	0.63	867.55	.651	.930
I tried and failed to cut down on or stop eating certain foods	1.939	1.700	0.61	871.49	.648	.930
I was so distracted by eating that I could have been hurt (e.g.**,** driving a car, crossing the street, operating machinery)	1.465	1.280	0.61	895.93	.546	.931
I was so distracted by thinking about food that I could have been hurt (e.g.**,** driving a car, crossing the street, operating machinery)	1.370	1.036	0.57	904.71	.540	.932
My friends and family were worried about how much I overate	1.748	1.689	0.68	873.24	.634	.930

*M*  mean, *SD*  standard deviation, *FL*  factor loadings, *V*  scale variance if item deleted, *I.T.*  corrected item-total correlations, *C.D.*  Cronbach’s alpha if item deleted

**Fig. 1:**
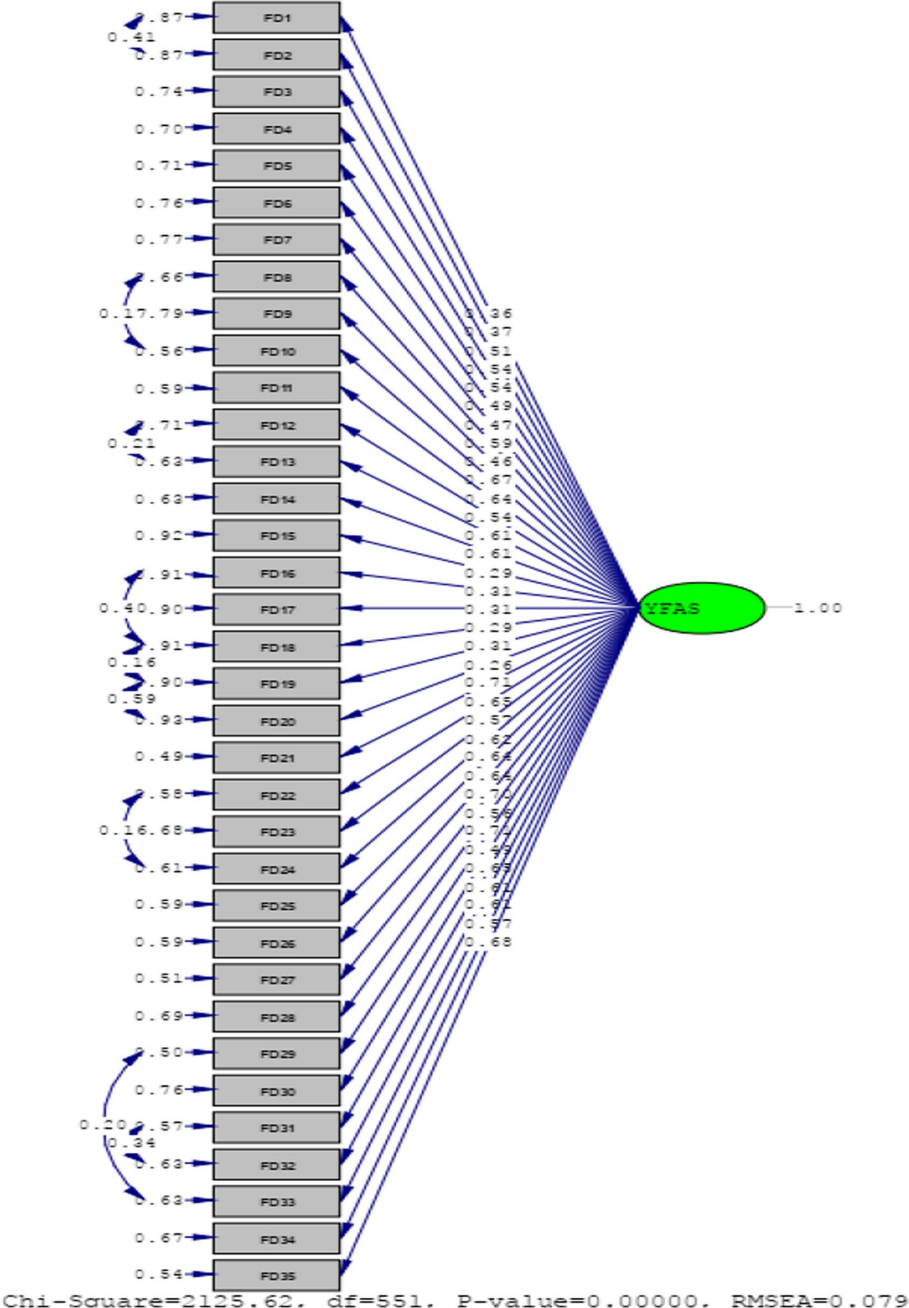
Results of confirmatory factor analysis of the Yale Food Addiction Scale Version 2.0 (YFAS 2.0)

Confirmatory factor analysis displayed that single-factor structure provided a good fit to the data: _sb_X^2^ = 2125.62 (*p* < 0.001); SRMR = 0.078; *CFI* = 0.94; *NFI* = *0.92; PNFI* = *0.85; IFI* = *0.94; RFI* = *0.91; GFI* = 0. 90; RMSEA = 0.078. All items of loads show a significant factor (Tables 2, 3); (Fig. 1).

**Table 3:** Model fit index

_sb_X^2^	SRMR	CFI	NFI	PNFI	IFI	RFI	GFI	RMSEA
2125.62**	0.078	0.94	0.92	0.85	0.94	0.91	0.90	0.078

*SRMR*  standardized root mean square residual, *CFI*  comparative fit index, *NFI* normed fit index, *PNFI* parsimony normed fit index, *IFI* incremental fit index, *RFI*  relative fit index, *GFI* comparative fit Index, *RMSEA*  root mean square error of approximation

**P ≤ 0.001

### Content validity ratio (CVR)

Experts were asked to review each item for determining the *CVR* based on a three-part range of “necessary”, “useful but not necessary,” and “not necessary”. Then the *CVR* was calculated according to the following Lawshe’s formula [23]:

$$CVR = \frac{{n_{e} - N/2}}{N/2}$$

In this regard, *n*_*e*_ is the number of specialists who have answered the necessary option, and *N* is the total number of specialists. If the calculated value is greater than the table value, its content validity is accepted and numbers above 0.60 were obtained [23].

### Content validity index (CVI)

Three criteria of simplicity, specificity, and clarity separately in a spectrum, 8-part Likert for each item was examined to examine the *CVI* by ten experts. They aggregated the scores for each item that scored 3 and 4 (highest score) calculated on the specialists’ total number; (1) Unrelated, (2) Somewhat related, (3) Related, (4) Completely related. Then, the average *CVI* was calculated for all the questionnaire questions. According to the number obtained about the questionnaire’s validity, the decision criteria were used to assess the *CVI* [24].

Unacceptable: *CVI* < 0.7.

The questionnaire needs to be modified and revised: 0.79 < *CVI* < 0.7

Acceptable *CVI* > 0.79.

### Face validity

This study evaluated its appropriateness, representativeness, readability, and clarity using face validity. Evaluation of surveys can be accomplished in a variety of ways. The most common method is cognitive interviews. Before collecting data from a large sample, cognitive interviews are helpful for researchers to clarify the items, ensure adequate content coverage, and modify the questionnaire if any questions are unclear [25]. Thirteen college students participated in cognitive interviews to determine item complexity, vagueness, and comprehensibility of interview items. Thirty-five questions were ultimately compiled as a final scale. As a result, no changes had to be made to the Persian scale when designing the YFAS 2.0's final Persian translation, as there were no unclear Persian terms.

### Internal consistency reliability

The internal consistency reliability of the Iranian Yale Food Addiction Scale Version 2.0 (YFAS 2.0) was assessed using Cronbach’s alpha for all participants and was 0.93.

### Follow-up study and test–retest reliability

For temporal stability analysis using a test–retest strategy after two weeks, 120 people were randomly contacted from the total sample, including 451. They were asked to complete the YFAS 2.0 again, and 112 people completed and sent it. The results showed that after this period, the calculated test–retest coefficient was 0.85(CI = 0.79–0.83).

### Convergent validity

The correlations between the Yale Food Addiction Scale Version 2.0 (YFAS 2.0) and the components of the Food Craving Questionnaire-Trait, reduced (FCQ-T-r), and DASS-21 indicate convergent validity. There was a significant positive correlation between YFAS 2.0 and factors of FCQ-T-r; [desire (*r* = 0.71; *P* < 0.001), hunger (*r* = 0.58; *P* < 0.001), reinforcement (*r* = 0.69; *P* < 0.001)] and significant positive relationship between YFAS 2.0 with factors of DASS-21; [DASS-21; depression (r = 0.33; *P* < 0.01), DASS-21 stress (r = 0.30; *P* < 0.01), DASS-21 anxiety (r = 0.39; *P* < 0.01) and DASS-21 total score (r = 0.38; *P* < 0.01)]. These findings illustrate acceptable convergent validity (Table 4).

**Table 4:** Person Correlation between the Yale Food Addiction Scale Version 2.0 (YFAS 2.0) with the components of Food Craving Questionnaire-Trait, Reduced (FCQ-T-r), and DASS-21 in the student sample

	M(SD)	1	2	3	4	5	6	7	8	9
1. YFAS 2	64.21(30.34)	1								
2. Desire	11.44(6.17)	.71**	1							
3. Hunger	6.73(3.74)	.58**	.75**	1						
4. Reinforcement	11.81(6.33)	.69**	.85**	.77**	1					
5. FCQ-T-r total	29.98(15.18)	.72**	.95**	.87**	.95**	1				
6.DASS-21-Depression	12.36(4.53)	.33**	.37**	.38**	.37**	.40**	1			
7.DASS-21-Stress	13.71(4.51)	.30**	.36**	.38**	.38**	.39**	.74**	1		
8.DASS-21-Anxiety	11.61(3.81)	.39**	.41**	.40**	.39**	.43**	.66**	.65**	1	
9.DASS-21-total	37.69(11.44)	.38**	.42**	.44**	.42**	.46**	.91**	.90**	.85**	1

YFAS 2.0: Yale Food Addiction Scale Version 2.0; FCQ-T-r: Food Craving Questionnaire-Trait, Reduced

*P ≤ 0.05

**P ≤ 0.01

## Discussion

The present study evaluated the psychometric assessment of the Persian translation of Yale Food Addiction Scale Version 2.0 (YFAS 2.0) in Iranian college students. The confirmatory factor analysis showed that each item had a suitable factor load and the scale had a good fit, confirming the one-dimensional structure of the original YFAS 2.0. In this study, standardized factor loadings were significant, which indicates the ability to distinguish items on this scale [26]. So we confirmed that the Persian translation of YFAS 2.0 is a useful tool for assessing food addiction among college students. As expected, moderate to high correlations were found between YFAS 2.0 and the DASS-21 and Food Craving subscales components, indicating YFAS 2.0 has high convergent validity that is consistent with previous research [26, 27]. A study of 1349 college students showed that psychological distress, such as anxiety, stress, and depression, was significantly negatively correlated with the symptoms of food addiction [28]. Psychological distress is strongly associated with food addiction and unhealthy eating habits, such as a lack of vegetables and a high intake of high-calorie foods.

The first step in diagnosing and treating food addiction is to evaluate it. The scale of food addiction due to its psychometric benefits such as execution and scoring of lead and a multidimensional evaluation of this variable can help therapists in this field [29, 30]. According to the appropriate psychometric properties of this scale in the present study, it can be stated that this scale can be widely used for measuring food addiction. In general, despite the appropriate validity coefficients of the Persian translation of the Yale Food Addiction Scale Version 2.0, ease of implementation and conditions of use in different situations and groups enable researchers to use this scale extensively in various research fields of clinical psychology.

It is worth noting that the limitations of the present study should be considered in addition to this scale’s strengths. First, participants were selected from the university level. This sample may not represent the general population; therefore, it is recommended that subsequent research be conducted with samples that represent the general population. Research can even be conducted with clinical populations to evaluate the discriminant validity of the scale. Second, the present study was conducted with self-report data. This data is inherently subject to bias. It is also suggested that future studies examine the differences between food addiction between men and women. Finally, based on the present study’s findings, it can be acknowledged that the Persian translation of Yale Food Addiction Scale Version 2.0 (YFAS 2.0) with appropriate psychometric properties has the conditions of use in college students.

It seems that further structural questions (e.g., diet status) should be used in future research to assess changes in diet and lifestyle behaviors over time. It is considering studies have shown that drug addiction and food addiction have some clinical, neurological, psychiatric, and socio-cultural risk factors. The relationship between environmental effects, such as availability and exposure to certain types of food, can also be examined in future research. It is recommended to conduct similar studies in various samples with obesity and weight loss problems and compare individual differences. Since the measurement of food addiction is essential, it is suggested that research be conducted on a larger scale to provide the generalizability of the results and reflect the general population. It is recommended that studies be performed on patients with obesity, metabolic syndrome, type 2 diabetes, hypertension, dyslipidemia, atherosclerosis, stroke, or cardiovascular disease who are at risk for complications related to food addiction. This research can pave the way for researchers and nutritionists. Through this study, we hope to provide valuable tools to measure addictive-like eating behavior in the community, clinical, and research contexts through the Persian translation of the YFAS 2.0.

## Conclusion

Addictive-like eating behaviors are a significant issue among individuals, causing many physical problems such as cardiovascular disease, type 2 diabetes, cancer, and rising mortality and treatment costs. This is the first time this questionnaire has been validated in Iranian college students. Therefore, researchers and specialists can use this scale for diagnosing food addiction for research or diagnostic purposes in Iranian college students. In conclusion, the YFAS 2.0 showed good psychometric properties in assessing food addiction among Iranian college students.

## Data Availability

The datasets generated during and analyzed during the current study are available from the corresponding author upon reasonable request.
